# Boron and pyridinic nitrogen-doped graphene as potential catalysts for rechargeable non-aqueous sodium–air batteries†

**DOI:** 10.1039/d0ra03126g

**Published:** 2020-06-09

**Authors:** Natei Ermias Benti, Girum Ayalneh Tiruye, Yedilfana Setarge Mekonnen

**Affiliations:** Center for Environmental Science, College of Natural and Computational Sciences, Addis Ababa University P. O. Box 1176 Addis Ababa Ethiopia yedilfana.setarge@aau.edu.et; Materials Science Program/Department of Chemistry, College of Natural and Computational Sciences, Addis Ababa University P. O. Box 1176 Addis Ababa Ethiopia

## Abstract

In this work, we performed density functional theory (DFT) analysis of nitrogen (N)- and boron (B)-doped graphene, and N,B-co-doped graphene as potential catalysts for rechargeable non-aqueous sodium–air batteries. Four steps of an NaO_2_ growth and depletion mechanism model were implemented to study the effects of B- and N-doped and co-doped graphene on the reaction pathways, overpotentials, and equilibrium potentials. The DFT results revealed that two-boron- and three-nitrogen (pyridinic)-doped graphene exhibited plausible reaction pathways at the lowest overpotentials, especially during the charging process (approximately 200 mV), thus, significantly improving the oxygen reduction and oxidation reactions of pristine graphene. In addition, pyridinic nitrogen-doped graphene meaningfully increased the equilibrium potential by approximately 0.30 eV compared to the other graphene-based materials considered in this study. This detailed DFT study provides valuable data that can be used for the successful development of low-cost and efficient graphene-based catalysts for sodium–air battery systems operating with non-aqueous electrolyte.

## Abstract 2

We performed density functional theory analysis of heteroatom doped graphene as potential catalysts for rechargeable non-aqueous sodium–air batteries. Pyridinic nitrogen and boron doped graphene exhibited too low overpotential reaction pathways.

## Graphical abstract

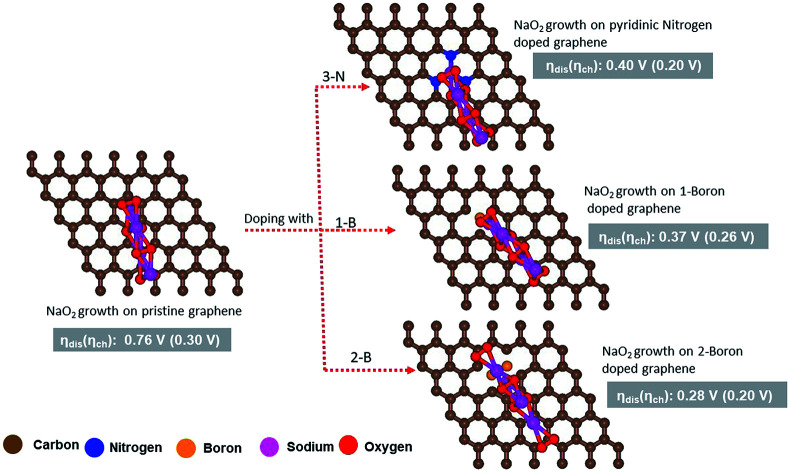

## 1. Introduction

In an attempt to make batteries viable for use in future transportation, there has been significant research and development of rechargeable metal–air batteries (Li–, Na– and Zn–O_2_/air batteries).^1–7^ A sodium–air battery (Na–O_2_) consists of sodium metal as the anode and an air/oxygen cathode in which environmental oxygen can be used. Recently, there has been considerable interest in Na–O_2_ due to its relatively high energy density and capacities, operation at low dis/charge overpotentials, high electrical energy efficiency (approximately 90%), and operation over multiple cycles with chemical reversibility comparable to that of lithium ion batteries.^8–12^ Most importantly, due to the abundance of sodium in the Earth's crust (approximately 2.6% by weight, nearly 4–55 orders higher than that of lithium), the cost of a rechargeable sodium–air battery is the lowest compared with other battery technologies (Fig. S1†).^13^ It is preferable to use low-cost rechargeable sodium–air batteries in electric vehicles (EVs) and hybrid electric vehicles (HEVs) to compete with conventional automobiles built with internal combustion engines.^13,14^

A non-aqueous sodium–air battery system can be constructed by combining a porous carbon material as an air/oxygen cathode and pristine sodium metal as an anode material. In the discharge process, NaO_2_ or Na_2_O_2_ can be produced by the reaction of O_2_^−^ or O_2_^2−^ at the air cathode with that of Na^+^ produced from the sodium anode. These solid discharge products (NaO_2_/Na_2_O_2_) can accumulate onto the air cathode material and easily clog the diffusion pathways, resulting in the deterioration of Na–O_2_ battery performance due to the steric barrier for Na^+^/O_2_ diffusion.^13^ In addition, during the charging process of the Na–O_2_ battery, the polarization is increased due to the over-accumulation of insoluble discharge products, which have a very low electrical conductivity, and thus tend to exhibit sluggish reaction kinetics during the oxygen reduction reaction (ORR) and oxygen evolution reaction (OER).^15^

Therefore, development of inexpensive, stable, and highly efficient air cathode materials with high catalytic efficiency for the OER and ORR is of high importance for the development of efficient non-aqueous rechargeable Na–O_2_ batteries.^15–19^ Different air cathode materials have been used to improve the sluggish reaction kinetics. These include carbon-based,^16,18–23^ noble metals,^24–26^ metal oxides,^27–31^ and metallic alloys,^32,33^ and they are among the numerous proposed potential catalysts for ORR and OER. In particular, carbon-based materials are advantageous for such uses due to their large specific surface area, high conductivity, and low cost.

Graphene, a two-dimensional nanomaterial with sp^2^-bonded carbon atoms, is widely used as a cathode material in metal–air batteries due to its extraordinary properties such as lightest weight (approximately 2 g cm^−3^), ultrahigh theoretical surface area (2630 m^2^ g^−1^),^34,35^ extremely high chemical stability,^34,36^ and superior electronic conductivity and mechanical strength (100–300 times higher than steel).^37,38^ However, its application in various energy-related devices is limited due to the unusual semi-metallic nature of graphene with its zero band gap at the Dirac point.^39^ Although some methods exist for opening the zero band gap in graphene,^40–42^ heteroatom doping has proved to be effective in improving the semiconducting properties of graphene. The dopant atom modifies its electronic band structure and opens up an energy band gap between the valence and conduction bands.^16–19,43–46^

There has been great interest in nitrogen- and boron-doped graphene sheets due to the comparable atomic sizes between N and B and the C atom and their strong probability of entering a graphene lattice and forming p-type and n-type semiconducting graphene, which allows the fabrication of modern graphene-based electrochemical energy storage materials.^47–52^ Experimentally, it has been proved that graphene nanosheets (GNSs) can act as potential cathode materials in Na–air batteries, with a high discharge capacity at a high current rate and stable cycling behavior that was maintained up to 10 cycles.^53^ In another study by Li *et al.*^45^ nitrogen-doped graphene nanosheets (N-GNSs) were studied as a cathode material in Na–O_2_ batteries. They reported that the N-GNSs exhibited more optimal electrocatalytic activity for the ORR and lower overpotentials than pristine GNS. Additionally, the results also revealed that the discharge capacities of N-GNSs were twice as much as those of pristine GNSs at all considered current densities.^45^ The experimental results showed that the synergistic effect of N and B co-doped graphene materials also increased the cathodic current in acidic and basic medium,^54–58^ proving enhanced performance of N,B co-doped graphene towards the ORR process.

Apart from experimental studies, the computational insights into ORR/OER are also a useful tool to carefully investigate the catalytic mechanism in non-aqueous metal–O_2_ batteries. A recent computational analysis of non-aqueous Li–O_2_ batteries indicated that heteroatom-doped graphene can significantly reduce the activation barrier of O_2_ evolution in the OER pathway.^16–19^ Among the five N-doped graphene configurations studied by Jing and Zhou,^17^ in-plane pyridinic N-doped graphene exhibited a more optimal performance in the catalytic activity of the ORR process. A density functional theory (DFT) analysis also predicted that B-doped graphene can be a good catalyst in Li–air batteries by reducing the oxygen evolution barrier due to its p-type behavior.^19^

Recently, the ORR/OER performance of pristine graphene, N-, B-doped graphene, and N,B co-doped graphene was predicted using DFT for potential catalysts for the ORR/OER in non-aqueous Li–O_2_ batteries. It was found that B-doped graphene presents the lowest discharge and charge overpotentials compared with N,B co-doped graphene, indicating the best catalyst for both the ORR and the OER processes in Li–air batteries.^16^ Despite the experimental investigation of the performance of doped graphene in non-aqueous-based Na–O_2_ batteries, there is limited information regarding the theoretical prediction of ORR/OER mechanisms. Moreover, to the best of our knowledge, there is no theoretical information regarding the effect of doped graphene on the growth/depletion of NaO_2_, which is the main discharge product in Na–O_2_ batteries, on B- and N-doped surfaces and their co-doped graphene in Na–air batteries.

Herein, DFT calculations were employed to investigate the catalytic activity of pristine graphene, B- and N-doped graphene, and B,N co-doped graphene materials towards ORR/OER for an Na–O_2_ battery system operating with an aprotic electrolyte. The effect of doping heteroatoms (B and N) on graphene sheets at various doping concentrations on NaO_2_ growth and depletion pathways, overpotentials, and equilibrium potentials was studied. Moreover, the charge transfer properties of pristine, doped, and co-doped (B and N) graphene materials were investigated using Bader charge analysis. A 6 × 6 × 1 graphene supercell containing 72 carbon atoms was arranged to examine the catalytic effect of heteroatom doping in ORR/OER mechanism studies. The number of B and N dopant atoms were increased from one to two and one to three, respectively, in the graphene structure, and doping occurred by substituting some of the carbon atoms of the graphene. Furthermore, N,B-co-doped graphene structures with separated and bonded sites were also considered in this study. This detailed computational investigation on heteroatom (B and N)-doped and co-doped graphene will increase our understanding of the NaO_2_ growth and depletion (ORR/OER) processes in rechargeable non-aqueous Na–O_2_ batteries and will provide clues regarding the design of novel doped graphene-based catalysts for rechargeable non-aqueous sodium–air batteries.

## 2. Computational details

DFT^59,60^ calculations were performed with the grid-based projector-augmented wave method (GPAW) code^61,62^ through the atomic simulation environment (ASE).^63^ A real space grid basis set on the projector augmented wave (PAW) function method with frozen core approximation^64,65^ was used with 0.18 Å grid point spacing. Electron exchange and correlation was managed by the generalized gradient approximation (GGA) of the Perdew–Burke–Ernzerhof (PBE) functional.^66^ A 6 × 6 × 1 graphene supercell with 72 carbon atoms was employed for all structures considered in this study. Heteroatom (B and N) doping on graphene sheets was possible by substituting some of the carbon atom/s of the graphene sheet with the heteroatom/s of interest. The vacuum layer along the *z*-axis was set to be 20 Å to avoid any interlayer interactions in all calculations. The *k*-points were sampled with 4 × 4 × 1 Monkhorst–Pack grids, and 0.18 Å grid point spacing is used. Atomic energy optimization calculations were performed until all forces were less than 0.03 eV Å^−1^. The charge population was calculated by using Bader charge analysis. The stability of the chosen structures was estimated from the formation energies using the following equation:1
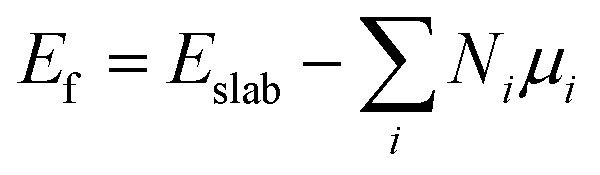
where *E*_f_ denotes the formation energy, *E*_slab_ denotes the DFT total energy calculated for the slab model, and *N*_*i*_ and *μ*_*i*_ represent the total number and chemical potential of each atom, respectively. The chemical potential of carbon (*μ*_C_) is defined as the total energy of graphene per carbon atom, and the chemical potential (*μ*_N_) of N is taken as one-half of the total energy of the N_2_ molecule in the gas phase. The chemical potentials of the other atoms were obtained from their bulk phases. According to eqn (1), the *E*_f_ of pristine graphene is naturally set to be zero and taken as the reference.

To identify a potential-limiting step, thermodynamic free energy diagrams were calculated for the ORR/OER in the graphene, B- and N-doped and their co-doped graphene surfaces as a function of electrode potential (*U*). Thermodynamic potentials for the charging and discharging processes were deduced by calculating free energies of all intermediates shown in the elementary reaction steps. It is assumed that Na^+^ + e^−^ are in electrochemical equilibrium at *U* = 0 V with a bulk Na metal. It is also assumed that the electrochemical potential of an electron shifts by −*eU* when electrode potential *U* initiates according to Nernst equation (*U*_0_ = Δ*G*/*ne*), where Δ*G* denotes the change in Gibbs free energy, *n* denotes the number of electrons involved with the electrochemical reaction, and *e* indicates the elementary charge. We defined overpotential for the discharging (charging) process as the maximum (minimum) potential to shift the free energies of all intermediates of ORR/OER downhill.

Large systematic errors in the description of superoxides, peroxides, and monoxides have previously been documented by various groups and have been accounted for in various ways.^67–69^ Here, we adopted the approach of Christensen *et al.*^69^ using NaCl as an indirect reference for sodium in order to better account for the oxidation state of Na in the Na–O_2_ system. This approach was chosen because it significantly reduces systematic errors while allowing consistent calculation of surfaces with oxide species in different oxidation states required for studying reactions in Na–O_2_ batteries. To compensate for the overestimation of the binding energy of O_2_ in the DFT calculation, in accordance with Christensen *et al.*,^69^ an energy correction was applied to O_2_ (−0.33 eV). Hence, the corrected energy per formula unit of O_2_ was calculated according to eqn (2):2
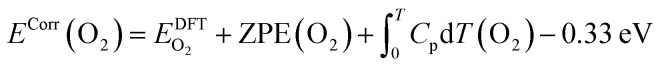
where *E*_O_2__^DFT^ denotes the calculated ground-state DFT energy, ZPE denotes the zero-point energy, and the integral is over the constant-pressure heat capacity of the O_2_ molecule.

The ORR/OER process was described by the adsorption/desorption of both electrochemical (involving either Na* or 
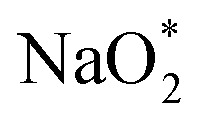
) and chemical (involving O_2_) species. In this study, only the thermodynamically favorable path (*i.e.*, the lowest overpotential path) was determined by comparing the free energies of (electro) chemical reaction steps. The free energy of the intermediates in each step was calculated using energy-corrected O_2_ and sodium chloride (NaCl) as references, and is thus given as:

3 Δ*G* = Δ*E* + ΔZPE − *T*Δ*S*

where Δ*G* denotes the calculated free energy difference, Δ*E*, ΔZPE, *T*, and Δ*S* indicate the DFT total energy difference, changes in zero-point energy, temperature, and entropy of the slab of intermediates adsorbed on the catalyst surface at 300 K, respectively. In the case of solid–solid reactions, the change in ΔZPE and Δ*S* is often small, and Δ*E* becomes the predominant contributor in the free energy. However, due to the adsorption of gaseous molecules on the surfaces of solids, the change in entropy can be significant, and contribution should be considered (*T*Δ*S* (O_2_) = 0.63 eV and ΔZPE (O_2_) = 0.131 eV).

The adsorption energies (*E*_ads_) of intermediates on the catalyst surfaces were calculated by the following equation:

4 *E*_ads_ = *E*(intermediate + substrate) − [*E*(intermediate) + *E*(substrate)]

where *E*(intermediate + substrate), *E*(intermediate), and *E*(substrate) refer to total energy of intermediate with a substrate, intermediate molecule, and a substrate alone, respectively.

## 3. Results and discussion

### 3.1 Geometric and electronic properties of doped graphene

**Fig. 1:**
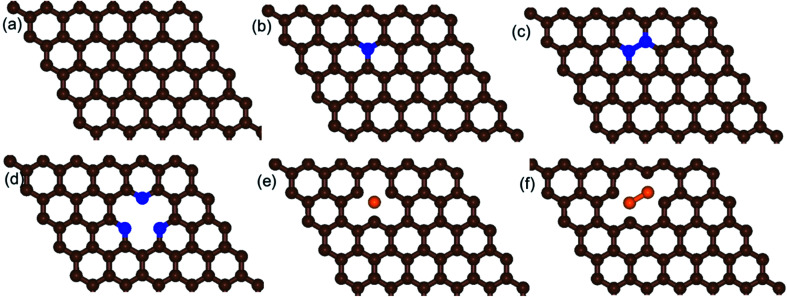
Schematic diagrams of the structures of (a) pristine graphene, (b) one-nitrogen-doped graphene (NG), (c) two-nitrogen-doped graphene (2NG), (d) three-nitrogen (pyridinic)-doped graphene (pyr-3NG), (e) one-boron-doped graphene (BG), and (f) two-boron-doped graphene (2BG). The brown, blue, and orange spheres indicate C, N, and B atoms, respectively.

We built a graphene sheet that contained one nitrogen and one-boron dopant atom, and it was explored for its ORR activities. Then, the number of nitrogen and boron dopant atoms were increased from one to three and one to two, respectively. The reason for increasing the number of nitrogen dopant atoms was to introduce the pyridinic three-nitrogen into the structure of graphene. The most stable structures for nitrogen- and boron-doped graphene sheets are shown in Fig. 1. The nitrogen-doped graphene (NG) and boron-doped graphene (BG) structures were built by replacing one C atom from a graphene structure with a N or B atom in a 6 × 6 supercell, corresponding to a N or B content of 1.39 wt%.

The electrical property and chemical reactivity can be effectively modified by the introduction of heteroatoms into the sp^2^-hybridized carbon framework of graphene.^16–19^ The formation energies calculated using eqn (1) above and average bond lengths between X–C (X = B or N) for the doped graphene structures are listed in Table 1.

**Table 1:** The formation energies (*E*_f_) of all doped graphene samples and their corresponding average bond lengths

System	Graphene	NG	BG	b-NBG	s-NBG	2NG	2BG	pyr-3NG
*E*_f_ (eV)	0.000	0.960	0.300	−1.090	0.168	1.696	1.537	3.316
Bond length (Å)	1.424 (C–C)	1.411 (N–C)	1.488 (B–C)	1.488 (B–C)	1.483 (B–C)	1.407 (N–C)	1.480 (B–C)	1.337 (N–C)
				1.397 (N–C)	1.403 (N–C)	1.450 (N–N)		

#### Nitrogen-doped graphene

The length of the N–C bond in the one-N-doped graphene was revealed to be 1.411 Å (Fig. 1b), slightly smaller that of C–C bond (1.424 Å) in pristine graphene (Fig. 1a). However, due to the electron-withdrawing nature of N, the other two C–C bonds of the C atom bonded to N are slightly stretched to 1.420 Å. The formation energy (*E*_f_) for the NG structure is increased from that of the pristine graphene. When the number of doped nitrogen atoms increased from one to two (the content of N is approximately 2.78 wt%) by replacing two C atoms from the graphene structure (Fig. 1c), the bond length of N–C was found to be 1.407 Å, which is shorter than that of the bond lengths of N–C in the structures of one-nitrogen-doped graphene (NG) and pristine graphene. However, the bond length of N–N was slightly increased to 1.450 Å, probably due to the repulsion of the lone pair of electrons presented in the two nitrogen atoms. Despite the decrease in the bond length of N–C in the two-nitrogen-bonded graphene (2NG), the *E*_f_ (1.696 eV) of 2NG was increased from that of NG (see Table 1).

In addition, when the three nitrogen atoms (pyridinic nitrogen) were doped into the structure of graphene (pyr-3NG) (Fig. 1d), it revealed the shortest bond length of N–C (approximately 1.337 Å) compared with those in the NG and 2NG structures. This is probably due to the strongest electron withdrawing ability of the three nitrogen atoms, which can create instabilities in the structure.^17^ Recent studies showed that in-plane pyridinic N can also form tri-N substituted vacancies.^70–72^ With increasing numbers of N atoms, the *E*_f_ of pyr-3NG was also increased to 3.316 eV, which is higher than those in NG and 2NG structures. This highest *E*_f_ indicates that doping pyridinic nitrogen into the graphene structure resulted in an unstable pyr-3NG structure that will be more difficult to prepare than that of the NG and 2NG structures.^17^

#### Boron-doped graphene

Similarly, instead of a N atom, one and two boron atoms were separately doped into the structure of graphene (see Fig. 1e and f). The B–C bond length in the B-doped graphene (BG) structure was 1.488 Å, which is larger than the C–C bond in pristine graphene. The other two C–C bonds of the C atom bonded to B were shortened to 1.409 Å due to the electron-donating nature of B. When two boron atoms were doped into the structure of graphene (2BG), the B–C bond length was slightly shorter than that of the B–C in the BG structure. However, the *E*_f_ was higher in the 2BG structure (1.537 eV) as compared to that of the BG structure (0.300 eV), indicating that the formation of the 2BG structure is less favorable than that of the BG structure (see Table 1).

#### N,B-co-doped graphene

**Fig. 2:**
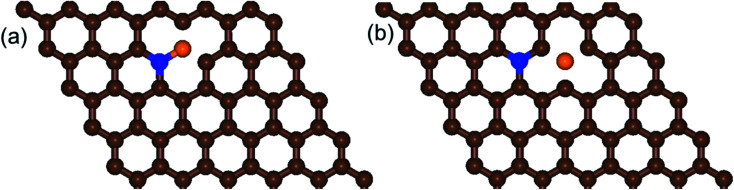
Schematic diagrams of the structures of (a) b-NBG and (b) s-NBG. The brown, blue, and orange spheres indicate C, N, and B atoms, respectively.

N,B-co-doped graphene was built with N and B directly bonded each other (b-NBG) and separated by a C atom (s-NBG) (see Fig. 2a and b). The N–C and B–C bond lengths in the b-NBG were approximately 1.397 and 1.488 Å, respectively. In the s-NBG structure, approximately 1.405 and 1.483 Å were obtained for N–C and B–C bond lengths, respectively. The N–C bond length in the b-NBG structure was higher than the same bond length in s-NBG. However, the change in the B–C bond length in b-NBG and s-NBG was insignificant.

Our calculation shows that the most stable B- and N-doped graphene and their co-doped graphene materials have a planar sp^2^ structure, which is in agreement with previous studies.^16,18^ As listed in Table 1, the decreasing order of formation energies of the catalysts is pyr-3NG > 2NG > 2BG > NG > BG > s-NBG > graphene > b-NBG. The formation energy for the b-NBG surface is negative (−1.09 eV), but the formation energy for the surface of the doped catalyst is positive (Table 1). This shows that the b-NBG is the dominant structure among all the possible catalysts. Therefore, co-doping of N and B atoms in the structure of graphene is more energetically favorable than single-atom doping. Moreover, due to the formation of B–N “parity” in the b-NBG structure, it is more stable than graphene, which has also been previously reported.^16^ The stability sequence for NG, BG, s-NBG, graphene, and b-NBG obtained in this work is in agreement with other research performed to obtain potential catalysts in non-aqueous Li–O_2_ batteries.^16^

#### Charge transfer analysis

The effectiveness of doped graphene-based materials as a catalyst for the ORR is closely related to its charge density distributions^73,74^ and the ability to activate sluggish carbon electrons for O_2_ utilization.^75,76^ Here, Bader charge density calculations were performed to determine how doped atoms influence electron distribution (Fig. S2†).

N-doped graphene structures (NG, 2NG, and pyr-3NG) showed negative electron transfer. The number of transferred negative electrons increased with increasing number of doped N atoms in the structure of graphene (Table 2). Due to the electron-withdrawing nature of the N atom, 0.48, 0.629, and 1.133 electrons were transferred from C atoms of the graphene sheet to the N atoms in the NG, 2NG, and pyr-3NG structures, respectively (Fig. S2b–d†). This indicates that as the number of N atoms increases, more active sites with a high-electron region will be created in the structure of the graphene sheet. The redistribution of charge density will also create active sites in graphene, a phenomenon that is beneficial for the ORR. Therefore, among the N-doped graphene sheets, more active sites are created around the doped atoms of pyr-3NG, and more optimal ORR performance is expected.

**Table 2:** The amount of charge transferred in the structures of doped graphene

Structure	Number of electrons transferred	Remark
Graphene	0	—
NG	−0.480	Transfer from the surface
2NG	−0.629	Transfer from the surface
pyr-3NG	−1.133	Transfer from the surface
BG	0.469	Transfer to the surface
2BG	1.458	Transfer to the surface
b-NBG	0.014	Transfer to the surface
s-NBG	0.565	Transfer to the surface

In B-doped graphene, approximately 0.47 electrons are transferred from B to C atoms of the graphene sheet (Table 2 and Fig. S2e†). In this case, due to the electron-donating nature of the B atom, it loses electrons. Similarly, the number of positive transferred electrons increased with increasing number of doped B atoms. As a result of introducing two B atoms to the graphene structure, approximately 1.458 electrons were transferred from B atoms to the C atom, creating more active carbon electrons for the ORR/OER process in the doped graphene sheet (Fig. S2f†). Similarly, 2BG will benefit from the ORR/OER process, due to its higher electron-donating nature than BG.

However, due to the neutralization of the vacant orbitals of B atoms by the lone-pair electrons from the N atom, high-electron density accumulation occurs between the N–B bonds in b-NBG. Therefore, b-NBG transfers the lowest number of electrons, indicating the creation of lower active sites in the doped structure and will result in unsatisfactory performance towards the ORR. However, when N and B atoms were co-doped and separated by the carbon, more electrons were transferred, indicating that more active sites are created around the doped atoms and more optimal ORR performance can be compared with b-NBG structure (Table 2); see Fig. S2h.† As a result, s-NBG is a better catalyst than single-atom doped structures (NG and BG) for the ORR process in the presence of protons.^75,76^ However, events can differ in the presence of sodium atoms. In general, it can be concluded that heteroatom dopants and increasing the number of dopants can improve the electronic structure of the graphene sheet *via* charge transfer and chemical reactivity.

#### Density of states (DOS)

**Fig. 3:**
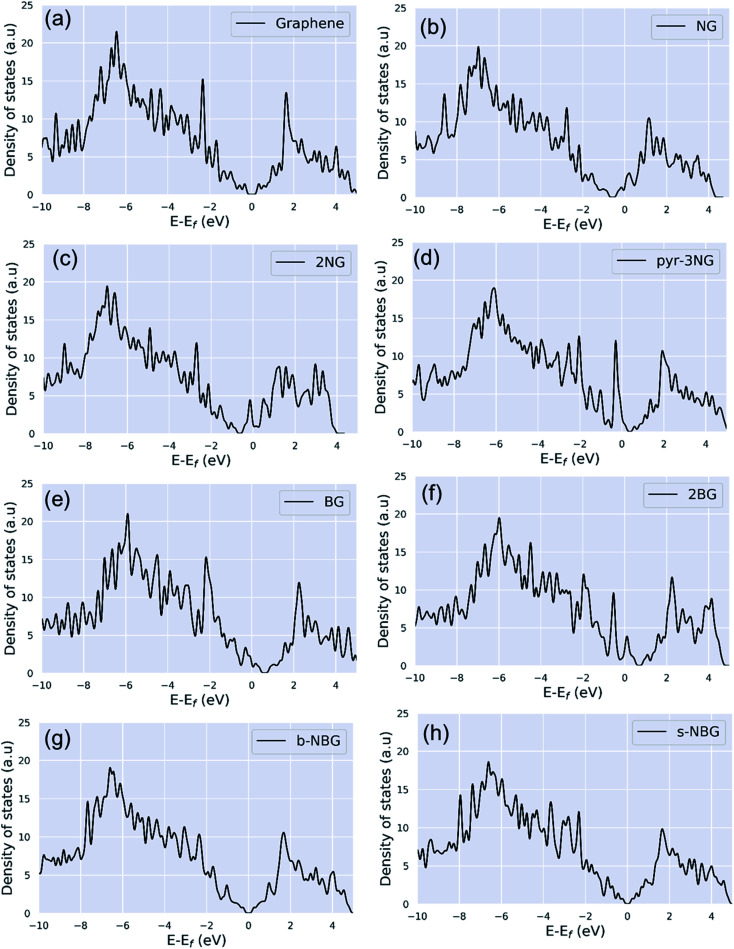
Density of states (DOS) of (a) pristine graphene, (b) NG, (c) 2NG, (d) pyr-3NG, (e) BG, (f) 2BG, (g) b-NBG, and (h) s-NBG.

For increased understanding of the change in the electronic structure caused by dopants, the total density of states (DOS) was calculated for pristine and all doped graphene configurations, as shown in Fig. 3. The DOS of the pristine graphene indicates that the valence and conduction band states touch each other at the Fermi level, demonstrating the zero-band-gap semiconducting characteristics (Fig. 3a).

N-doping graphene (NG and 2NG) can be regarded as an electron donor, which contributes one more electron to the delocalized π bond than the C atom. Thus, the Dirac points move towards the valance band below the Fermi level, and the DOS intensity near the Fermi level increases (Fig. 3b and c); thus, the formation energy increases compared to the pristine graphene. The number of electrons in the p_*z*_ orbital of nitrogen does not change in pyridinic nitrogen-doped graphene, but it causes a C vacancy, and the system loses one electron compared to pristine graphene. Thus, the system is like a p-doping semiconductor that shifts the Dirac point down. The DOS near the Fermi level is increased (Fig. 3d), and consequently, the formation energy is also increased. In B-doped graphene (BG and 2BG), the Dirac points shifted towards the conduction band (Fig. 3e and f), indicating the p-type semiconducting characteristic of these doped graphene sheets, in agreement with reported literature.^16,18^ This shifting of the Dirac points into unoccupied states can enhance the electrical conductivity of graphene after doping B with different concentrations. The p-type semiconducting characteristic results from the fact that the creation of some hole states around the Fermi level arises from the p orbitals of the B and C bonded to B. It is also noted that the DOS of co-doped graphene (b-NBG and s-NBG) shows semi-metallic behavior similar to that of pristine graphene (Fig. 3g and h). The conduction and valence bands meet near to the Fermi level, indicating that the band gap of the co-doped graphene is slightly less than that of the single atom-doped graphene.

### 3.2 NaO_2_ growth and depletion mechanisms on N-doped graphene

**Fig. 4:**
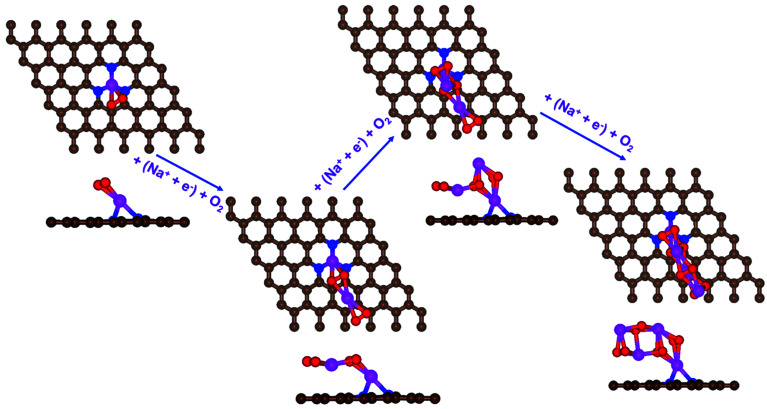
Schematic diagrams of the growing pathways of 4NaO_2_ on pyr-3NG. The brown and blue spheres indicate C and N atoms, respectively. Deposited atoms are colored as follows: Na, purple; and O, red.

**Fig. 5:**
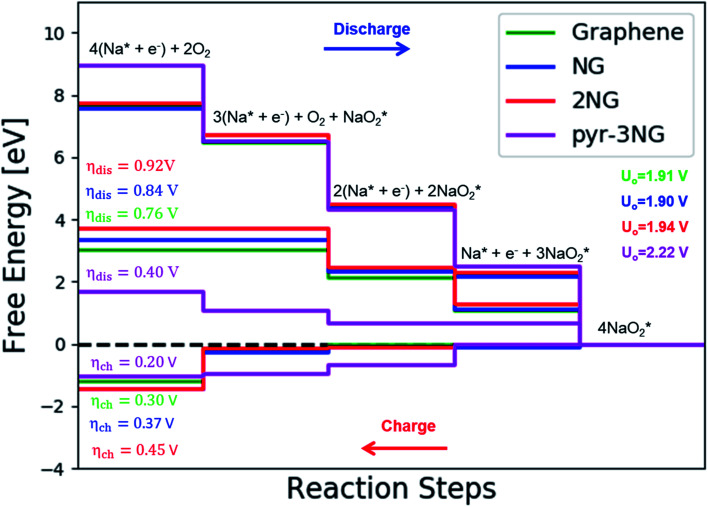
The calculated free energy diagram for a four-step growth mechanism for NaO_2_, with the most stable structures of intermediates on all nitrogen-doped graphene. The star-marked intermediates represent the adsorption states on the substrates.

First, a graphene cluster that contains one nitrogen dopant atom was built and explored to determine the ORR process. Then, the number of nitrogen dopant atoms was increased from one to three. Reversible potentials of ORR sub-reactions on these doped graphene sheets were calculated and compared each other. For comparison, pristine graphene sheet with the same configuration was also constructed. To generate entire reaction pathways and to determine the minimum theoretical overpotential, both electrochemical (involving either Na* or 
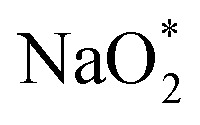
 species) and chemical (involving O_2_ species) reaction steps were considered. The thermodynamically favorable path (*i.e.*, the lowest overpotential path) was determined. The overpotential is used as an evaluation parameter to compare the catalytic effect of the substrates. As illustrated in Fig. 4, S3† and the free energy diagram in Fig. 5, we considered the following four-step reaction mechanisms to grow/deplete four formula units of NaO_2_ on the most stable doped graphene structures, *via* a series of four elementary steps:
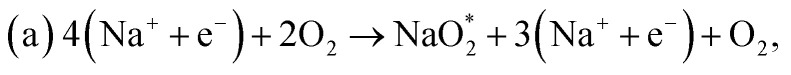

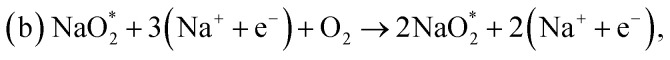

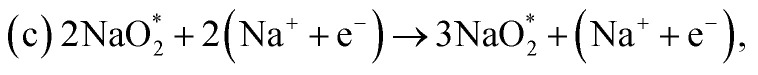

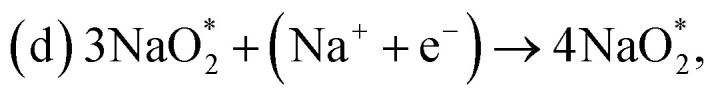
where * denotes adsorbate intermediate in a catalyst surface.

In the growth mechanism (ORR process), the first intermediate step was found to be the addition of a NaO_2_ specie for all of the N-doped graphene (Fig. 4 and S3†). This step was identified as the limiting discharge potential for pristine graphene, one-, and two-nitrogen-doped graphene (NG and 2NG). The reaction was followed by adding another NaO_2_ species, which is the limiting charge potential for 2NG. The third and fourth intermediate steps were also adsorption of two 
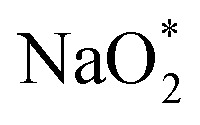
 species across the two NaO_2_ species previously adsorbed. For pristine graphene, pyr-3NG adsorption of the last intermediate was the limiting potential for the charging process, while adding the third NaO_2_ was the limiting discharge potential for pyr-3NG, and it was the limiting charge potential for NG.

The calculated free energy diagrams of the discharge process in pristine graphene, B,N-doped, and their co-doped graphene are all downhill at *U* = 0 V (from the left to the right, as shown in Fig. 5). In the discharging process, free energies remain downhill until the electrode potentials (*U*_dis_, which is a limiting discharge potential) reach 1.15 V, 1.06 V, 1.13 V, and 1.82 V in pristine graphene, NG, 2NG, and pyr-3NG, respectively. The catalytic effects of N-doped graphene are evaluated by overpotential, which is defined by *η*_dis_ = *U*_0_ − *U*_dis_ and *η*_ch_ = *U*_ch_ − *U*_0_ for the discharge and charge process, respectively. Hence, we identified each overpotential of the catalysts toward ORR in the discharging process as 0.76 V (pristine graphene), 0.84 V (NG), 0.92 V (2NG), and 0.40 V (pyr-3N), respectively, as shown in Fig. 5. The pyr-3NG-doped graphene exhibited the lowest ORR among the three N-doped graphene materials considered in this study. The charging process proceeded in the reverse direction of the free energy diagram for the discharge process, as shown in Fig. 5. Therefore, we characterized the OER as 0.30 V (pristine graphene), 0.37 V (NG), 0.45 V (2NG), and 0.20 V (pyr-3N), indicating that pyr-3NG exhibits the lowest overpotential toward the OER, and similarly, in the ORR. The overall reaction equilibrium potential is 1.91, 1.90, 1.94, and 2.22 V for pristine graphene, NG, 2NG, and pyr-3NG, respectively. This result shows that pyridinic N-doped graphene remarkably increased the equilibrium potential by approximately 0.30 eV compared to pristine graphene, NG, and 2NG. Thus, this study revealed that pyridinic nitrogen-doped graphene is a potential catalyst towards the ORR for rechargeable non-aqueous sodium–air batteries.

To further clarify the relationship between dis/charge limiting potential and *η*_dis_/*η*_ch_, the adsorption energy of intermediates in each dis/charge limiting potential were calculated, and the *η*_dis_/*η*_ch_ was plotted as a function of adsorption energy (Fig. S6†). As mentioned above, the limiting discharge potential for all N-doped catalysts is the formation of NaO_2_, except for pyr-3N (formation of Na_3_O_6_), where the adsorption energy of NaO_2_ on the catalysts is the determining factor for the *η*_dis_. The larger the adsorption energy, the more difficult it is for NaO_2_ to be involved in the subsequent reaction step, corresponding to a higher discharge overpotential. As can be seen in Fig. S6a,†*η*_dis_ increases as the adsorption energy increases, following the order of pristine graphene < NG < 2NG < pyr-3NG. Even if the limiting discharge potential of pyr-3NG is the formation of Na_3_O_6_ or addition of the third NaO_2_ species, its adsorption energy is less than that of pristine graphene, NG, and 2NG.

Similarly, for pristine graphene and pyr-3N, depletion of Na_4_O_8_ is the limiting charge potential, and the *η*_dis_ also shows a positive correlation with the adsorption energy of Na_4_O_8_, as shown in Fig. S6b.† Because the limiting charge potential of NG and 2NG is different from that of pristine graphene and pyr-3NG, we are unable to evaluate the relationship between their limiting charge potential and charge overpotentials. In general, the result revealed that the adsorption energy of intermediates in the dis/charge limiting potential significantly affects the *η*_dis_/*η*_ch_ through affecting its next step reaction. Moreover, the lower adsorption energy in dis/charge limiting potential has a positive effect on decreasing the *η*_dis_/*η*_ch_. The relationship between *η*_dis_/*η*_ch_ and charge transferred was also evaluated by calculating the charge transfer of the intermediates after the growth mechanisms were completed, using Bader charge analysis. As shown in Fig. S7,† the *η*_dis_/*η*_ch_ as a function of charge transfer plot revealed that the larger the amount of charge transferred from the adsorbent (Na_4_O_8_) to the N-doped graphene surfaces, the less dis/charge overpotential they possess.

### 3.3 NaO_2_ growth and depletion on B-doped graphene

**Fig. 6:**
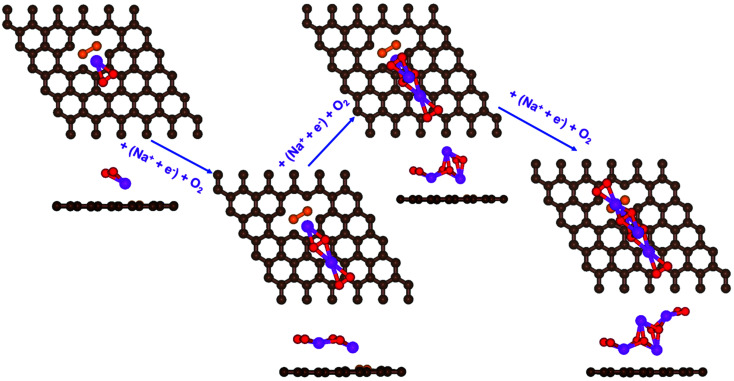
Schematic diagrams of the growing pathways of 4NaO_2_ on two B-doped graphene with a bonded configuration. The brown and orange spheres indicate C and B atoms, respectively. Deposited atoms are colored as follows: Na, purple; and O, red.

**Fig. 7:**
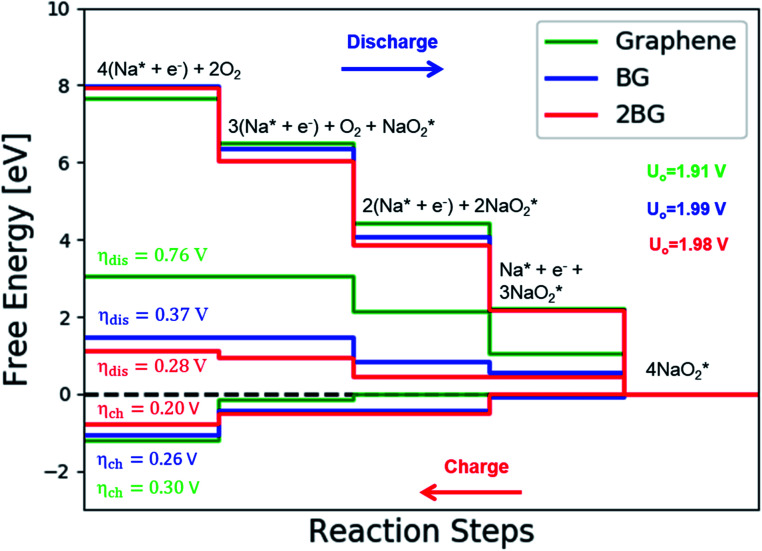
Calculated free energy diagram for a four-step growth mechanism for NaO_2_, with the most stable structures of intermediates on BG and 2BG. The star-marked intermediates represent the adsorption states on the substrates.

The catalytic effect of single- and two-boron-doped graphene on the ORR/OER process for the Na–O_2_ battery system was computationally investigated. As illustrated in Fig. 6, S4† and the free energy diagram in Fig. 7, the model follows a four-step reaction mechanism, and all reaction steps were found to be electrochemical. Four 
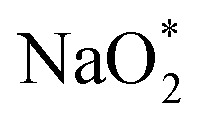
 species were successively adsorbed to the B-doped graphene surface to complete the growth mechanism during the discharge process. Addition of the first NaO_2_ species on all B-doped graphene surfaces was found to be the limiting discharge potential. Adding the second and third NaO_2_ was the potential limiting step for the charging process for BG and 2BG, respectively. The discharge overpotentials of BG and 2BG were identified as 0.37 V and 0.28 V, respectively (Fig. 6), indicating that both are suitable ORR catalysts in non-aqueous Na–O_2_ batteries. Again, the charging process follows the same reaction steps as the discharging process but in reverse order (right to left in Fig. 6). The charge overpotentials are characterized as 0.26 V and 0.20 V for BG and 2BG, respectively (Fig. 7), indicating that both are also active towards the OER. The overall reaction equilibrium potential is nearly the same, which is 1.99 V and 1.98 V for BG and 2BG, respectively.

As shown in Fig. S8,† dis/charge limiting potential and overpotentials (*η*_dis_/*η*_ch_) have a direct relationship. NaO_2_ is the limiting discharge potential for BG, while the limiting discharge potential for 2BG is Na_3_O_6_. The larger the adsorption energy, the greater the difficulty for NaO_2_/Na_3_O_6_ to be involved in the subsequent reaction step, corresponding to a higher discharge overpotential. To understand the underlying physical nature of the catalytic effect of B-doped graphene on the ORR/OER in a Na–O_2_ battery, Bader charge analysis was also performed. Table S3 and Fig. S9† display the transferred charge from cluster to substrate on both BG and 2BG. This shows that a significant amount of charge was transferred from the cluster to the substrate in BG and 2BG, which corresponds to lower dis/charge overpotentials as compared to pristine graphene. It indicates that charge transfer from Na_4_O_8_ to catalysts plays an important role in reducing dis/charge overpotentials. The charge transferred from Na_4_O_8_ to catalysts increases in the sequence of pristine graphene (0.1) < BG (0.45) < 2BG (0.50), indicating that 2BG is most active toward the ORR/OER as compared to pristine graphene and BG. However, relative to N-doped graphene clusters, both B-doped graphene exhibit lower dis/charge overpotentials. In our model, one and two of 72 C were replaced by one and two B atoms, and it is likely that a high-percentage doping will be more effective.

### 3.4 NaO_2_ growth and depletion mechanisms on N,B-co-doped graphene

**Fig. 8:**
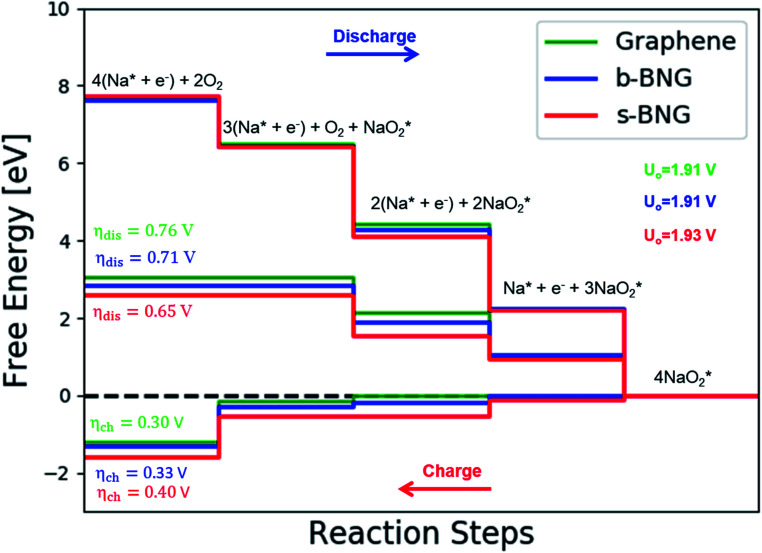
Calculated free-energy diagram for a four-step NaO_2_ growth mechanism with the most stable structures of intermediates on N,B-co-doped graphene with bonded and separated configurations.

Co-doped graphene with N and B bonded to each other (b-NBG) and separated by a C atom (s-NBG) was evaluated for its catalytic performance towards ORR/OER for non-aqueous Na–O_2_ batteries. Similarly, four-step reaction mechanisms were considered, and all reaction steps were found to be electrochemical, which is the successive addition of four NaO_2_ species to the doped graphene surfaces to complete the growth mechanism (Fig. 8 and S5†). Unlike the satisfactory performance of s-NBG and b-NBG in the proton-involved ORR process, the activity was lost due to the existence of sodium atoms, suggesting their poor electrocatalytic effect in non-aqueous Na–O_2_ batteries. The dis/charge overpotentials of both b-NBG and s-NBG co-doped structures were found to be 0.71/0.33 V and 0.65/0.40 V, respectively, with no significant change relative to pristine graphene. The charge transferred from Na_4_O_8_ to catalysts, and the dis/charge overpotentials also were positively correlated (Table S4†). Moreover, our DFT results partly confirmed the previous experimental reports that state that the synergistic effect of the combination of two different heteroatoms (B,N-co-doped graphene) exhibited improved performance towards the ORR process.^54–58^

**Fig. 9:**
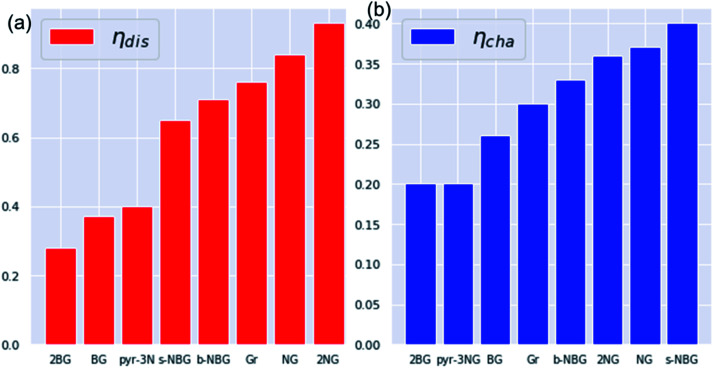
The calculated overpotentials of pristine and heteroatom-doped graphene materials in ascending orders showing (a) discharge overpotentials and (b) charge overpotentials.

The discharge overpotential of the catalysts toward the ORR in the discharging process was identified (Fig. 9), which increases in the sequence of 2BG < (0.28 V) < BG (0.36 V) < pyr-3NG (0.40 V) < s-NBG (0.65 V) < b-NBG (0.71 V) < graphene (0.76 V) < NG (0.84 V) < 2NG (0.92 V). The 2BG, BG, and pyr-3NG showed the lowest overpotential for ORR among the materials. The charge overpotential increases in the sequence of 2BG = pyr-3NG (0.20 V) < BG (0.26 V) < graphene (0.30 V) < b-NBG (0.33 V) < 2NG (0.34 V) < NG (0.37 V) < s-NBG (0.40 V), indicating that both B-doped graphene (BG and 2BG) and pyr-3NG are also suitable OER catalysts in non-aqueous Na–O_2_ batteries. As one can see from Fig. 9, the performance of 2NG and NG is even less than that of the pristine graphene towards ORR.

## 4. Conclusions

Density functional theory analysis was employed to investigate the catalytic activities of B-doped, N-doped and B,N-co-doped graphene with varying dopant concentrations for the ORR and OER in rechargeable non-aqueous Na–O_2_ batteries. It was confirmed that both single- and double-boron-atom-doped graphene exhibited too low dis/charge overpotentials, indicating high catalytic activities towards both the ORR and OER processes compared to other doped graphene samples. Nevertheless, one- and two-nitrogen-doped graphene essentially did not exhibit improvement. Introducing pyridinic nitrogen (pyr-3NG) into the structure of graphene revealed a higher equilibrium potential and significantly boosted the catalytic activity for both the ORR and OER processes as compared to the other doped graphene sheets considered in this study. The findings of this research partly confirm previous reports, which state that a combination of two different heteroatoms (B,N-co-doped graphene) resulted in more optimal catalytic activity for the ORR and OER, and subsequently enhanced the battery performance compared to pristine graphene.

Our DFT results suggest the viability of boron and nitrogen (especially pyridinic nitrogen) doping in graphene sheets as potential catalysts for the ORR and OER in rechargeable non-aqueous Na–O_2_ batteries. The NaO_2_ growth and depletion study with two-boron and three-nitrogen (pyridinic)-atom-doped graphene revealed too low limiting discharge/charge overpotential reaction pathways for both the oxygen reduction and oxygen evolution reactions, which elucidates the future design of non-precious carbon-based efficient catalysts in rechargeable non-aqueous Na–O_2_ batteries.

## Conflicts of interest

There are no conflicts to declare.

## Supplementary Material

RA-010-D0RA03126G-s001

## References

[cit1] Lutz L., Yin W., Grimaud A., AlvesDallaCorte D., Tang M., Johnson L., Azaceta E., Sarou-Kanian V., Naylor A. J., Hamad S., Anta J. A., Salager E., Tena-Zaera R., Bruce P. G., Tarascon J. M. (2016). J. Phys. Chem. C.

[cit2] Linden D. and Reddy T. B., Handbook of Batteries, McGraw-Hill, Third edn, 2001

[cit3] Mekonnen Y. S., Christensen R., García-Lastra J. M., Vegge T. (2018). J. Phys. Chem. Lett..

[cit4] Lysgaard S., Christensen M. K., Hansen H. A., Lastra J. M. G., Poul N., Vegge T. (2018). ChemSusChem.

[cit5] Benti N. E., Mekonnen Y. S., Christensen R., Tiruye G. A., Garcia-lastra J. M., Vegge T. (2020). J. Chem. Phys..

[cit6] Mekonnen Y. S., Garcia-Lastra J. M., Hummelshøj J. S., Jin C., Vegge T. (2015). J. Phys. Chem. C.

[cit7] Mekonnen Y. S., Knudsen K. B., Mýrdal J. S. G., Younesi R., Højberg J., Hjelm J., Norby P., Vegge T. (2014). J. Chem. Phys..

[cit8] Hartmann P., Bender C. L., Vračar M., Dürr A. K., Garsuch A., Janek J., Adelhelm P. (2013). Nat. Mater..

[cit9] Das S. K., Lau S., Archer L. A. (2014). J. Mater. Chem. A.

[cit10] Zhao N., Li C., Guo X. (2014). Phys. Chem. Chem. Phys..

[cit11] Bender C. L., Hartmann P., Vrac M., Janek J. (2014). Adv. Energy Mater..

[cit12] Knudsen K. B., Nichols J. E., Vegge T., Luntz A. C., Mccloskey B. D., Hjelm J. (2016). J. Phys. Chem. C.

[cit13] Yin W., Fu Z. (2017). ChemCatChem.

[cit14] Van Noorden R. (2014). Nature.

[cit15] Hartmann P., Bender C. L., Sann J., Dürr A. K., Jansen M., Janek J., Adelhelm P. (2013). Phys. Chem. Chem. Phys..

[cit16] Jiang H. R., Zhao T. S., Shi L., Tan P., An L. (2016). J. Phys. Chem. C.

[cit17] Jing Y., Zhou Z. (2015). ACS Catal..

[cit18] Ren X., Wang B., Zhu J., Liu J., Zhang W., Wen Z. (2015). Phys. Chem. Chem. Phys..

[cit19] Ren X., Zhu J., Du F., Liu J., Zhang W. (2014). J. Phys. Chem. C.

[cit20] Xu Y., Shelton W. A. (2011). J. Electrochem. Soc..

[cit21] Yun K., Hwang Y., Chung Y. (2015). J. Power
Sources.

[cit22] Kang J., Yu J.-S., Han B. (2016). J. Phys. Chem. Lett..

[cit23] Tiruye G. A., Noz-Torrero D. M., Berthold T., Palma J., Antonietti M., Fechler N., Marcilla R. (2017). J. Mater. Chem. A.

[cit24] Lu Y., Xu Z., Gasteiger H. A., Chen S., Hamad-schifferli K. (2010). J. Am. Chem. Soc..

[cit25] Lim H., Song H., Gwon H., Park K., Kim J., Bae Y., Kim H., Jung S., Kim T., Kim Y. H. (2013). Energy Environ. Sci..

[cit26] Krishna G. D. P., Shelton W. A., Xu Y. (2012). J. Phys. Chem. Lett..

[cit27] Døbart A., Paterson A. J., Bao J., Bruce P. G. (2008). Angew. Chem..

[cit28] Zheng Y., Song K., Jung J., Li C., Heo Y., Park M., Cho M., Kang Y., Cho K. (2015). Chem. Mater..

[cit29] Zhu J., Ren X., Liu J., Zhang W., Wen Z. (2015). ACS Catal..

[cit30] Tan P., Shyy W., Zhao T. S., Zhu X. B., Wei Z. H. W. (2015). J. Mater. Chem. A.

[cit31] Yoon T. H., Park Y. J. (2012). Nanoscale Res. Lett..

[cit32] Kim B. G., Kim H., Back S., Nam K. W., Jung Y., Han Y., Choi J. W., Li-o M. (2014). Sci. Rep..

[cit33] Choi R., Jung J., Kim G., Song K., Kim Y.-I., Jung S. C., Han Y.-K., Song H., Kang Y.-M. (2014). Energy Environ. Sci..

[cit34] Syama S., Mohanan P. V. (2019). Nano-Micro Lett..

[cit35] Stankovich S., Dikin D. A., Dommett G. H. B., Kohlhaas K. M., Zimney E. J., Stach E. A., Piner R. D., Nguyen S. T., Ruoff R. S. (2006). Nature.

[cit36] Yang M., Wang L., Li M., Hou T., Li Y. (2015). AIP Adv..

[cit37] David L., Bhandavat R., Barrera U., Singh G. (2016). Nat. Commun..

[cit38] Kim H., Park K., Hong J., Kang K. (2014). Sci. Rep..

[cit39] Geim A. K. (2009). Science.

[cit40] Han M. Y., Zyilmaz B. O., Zhang Y., Kim P. (2007). Phys. Rev. Lett..

[cit41] Boukhvalov D. W., Katsnelson M. I. (2008). Phys. Rev. B: Condens. Matter Mater. Phys..

[cit42] Mak K. F., Lui C. H., Shan J., Heinz T. F. (2009). Phys. Rev. Lett..

[cit43] Lherbier A., Blase X., Niquet Y., Triozon F., Roche S. (2008). Phys. Rev. Lett..

[cit44] Wu M., Cao C., Jiang J. Z. (2010). Nanotechnology.

[cit45] Li Y., Yadegari H., Li X., Banis M. N., Li R., Sun X. (2013). Chem. Commun..

[cit46] Zhang X., Xia Z., Li H., Yu S., Wang S., Sun G. (2019). RSC Adv..

[cit47] Lee J. H., Kwon S. H., Kwon S., Cho M., Kim K. H., Han T. H., Lee S. G. (2019). Nanomaterials.

[cit48] Lazar P., Zboril R., Pumera M., Otyepka M. (2014). Phys. Chem. Chem. Phys..

[cit49] Wang Z., Xiao J., Li X. (2012). Solid State Commun..

[cit50] Mukherjee S., Kaloni T. P. (2012). J. Nanopart. Res..

[cit51] Chang C., Yin S., Xu J. (2020). RSC Adv..

[cit52] Saito N. (2019). ACS Appl. Nano Mater..

[cit53] Liu W., Sun Q., Yang Y., Xie J.-Y., Fu Z.-W. (2013). Chem. Commun..

[cit54] Wang S., Iyyamperumal E., Roy A., Xue Y., Yu D., Dai L. (2011). Angew. Chem., Int. Ed..

[cit55] Wang S., Zhang L., Xia Z., Roy A., Chang D. W., Baek J. B., Dai L. (2012). Angew. Chem., Int. Ed..

[cit56] Choi C. H., Chung M. W., Kwon H. C., Park S. H., Woo S. I. (2013). J. Mater. Chem. A.

[cit57] Zheng Y., Jiao Y., Ge L., Jaroniec M., Qiao S. Z. (2013). Angew. Chem., Int. Ed..

[cit58] Zhang S., Cai Y., He H., Zhang Y., Liu R., Cao H., Wang M., Liu J., Zhang G., Li Y., Liu H., Li B. (2016). J. Mater. Chem. A.

[cit59] Hohenberg P., Kohn W. (1964). Phys. Rev..

[cit60] Kohn W., Sham L. J. (1965). Phys. Rev..

[cit61] Enkovaara J., Rostgaard C., Mortensen J. J., Chen J., Dułak M., Ferrighi L., Gavnholt J., Glinsvad C., Haikola V., Hansen H. A. (2010). et al.. J. Phys.: Condens. Matter.

[cit62] Enkovaara J., Romero N. A., Shende S., Mortensen J. J. (2011). Procedia Computer Science.

[cit63] Bahn S. R., Jacobsen K. W. (2002). Comput. Sci. Eng..

[cit64] Blochl P. E. (1994). Phys. Rev. B: Condens. Matter Mater. Phys..

[cit65] Mortensen J. J., Hansen L. B., Jacobsen K. W. (2005). Phys. Rev. B: Condens. Matter Mater. Phys..

[cit66] Perdew J. P., Burke K., Ernzerhof M. (1996). Phys. Rev. Lett..

[cit67] Kang S., Mo Y., Ong S. P., Ceder G. (2014). Nano Lett..

[cit68] Hummelshøj J. S., Luntz A. C., Nørskov J. K. (2013). J. Chem. Phys..

[cit69] Christensen R., Hummelshøj J. S., Hansen H., Vegge T. (2015). J. Phys. Chem. C.

[cit70] Yu Y.-X. (2013). Phys. Chem. Chem. Phys..

[cit71] Ma C., Shao X., Cao D. (2012). J. Mater. Chem..

[cit72] Rangel E., Magana L. F., Sansores L. E. (2014). ChemPhysChem.

[cit73] Zhang L., Xia Z. (2011). J. Phys. Chem. C.

[cit74] Zhang L., Niu J., Li M., Xia Z. (2014). J. Phys. Chem. C.

[cit75] Zhao Y., Yang L., Chen S., Wang X., Ma Y., Wu Q., Jiang Y., Qian W., Hu Z. (2013). J. Am. Chem. Soc..

[cit76] Yang L., Jiang S., Zhao Y., Zhu L., Chen S., Wang X., Wu Q., Ma J., Ma Y., Hu Z. (2011). Angew. Chem., Int. Ed..

