# Supplementary material for: Boron and pyridinic nitrogen-doped graphene as potential catalysts for rechargeable non-aqueous sodium–air batteries
Source: RSC Adv. 2020 Jun 9;10(36):21387–98. doi: 10.1039/d0ra03126g (PMC9054368; doi:10.1039/d0ra03126g)
Supplement: RA-010-D0RA03126G-s001 [file RA-010-D0RA03126G-s001.pdf]

Electronic Supplementary Material (ESI) for RSC Advances.
This journal is © The Royal Society of Chemistry 2020

Supplementary information

Boron and Pyridinic Nitrogen Doped Graphene as Potential Catalysts in Rechargeable Non-Aqueous Sodium-Air Battery

Natei Ermias Benti[1], Girum Ayalneh Tiruye[2], Yedilfana Setarge Mekonnen[1,*]

[1]Center for Environmental Science, College of Natural and Computational Sciences, Addis Ababa University, P. O. Box 1176, Addis Ababa, Ethiopia

[2]Materials Science Program/Department of Chemistry, College of Natural and Computational Sciences, Addis Ababa University, P. O. Box 33658, Addis Ababa, Ethiopia

Corresponding author email: yedilfana.setarge@aau.edu.et

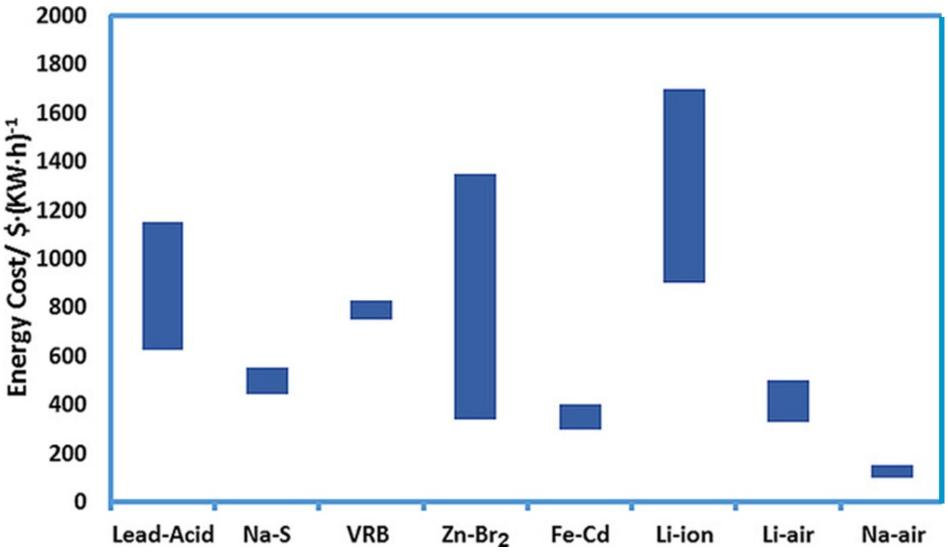

Figure S1: The energy cost of various batteries based on the current price of materials.[1]

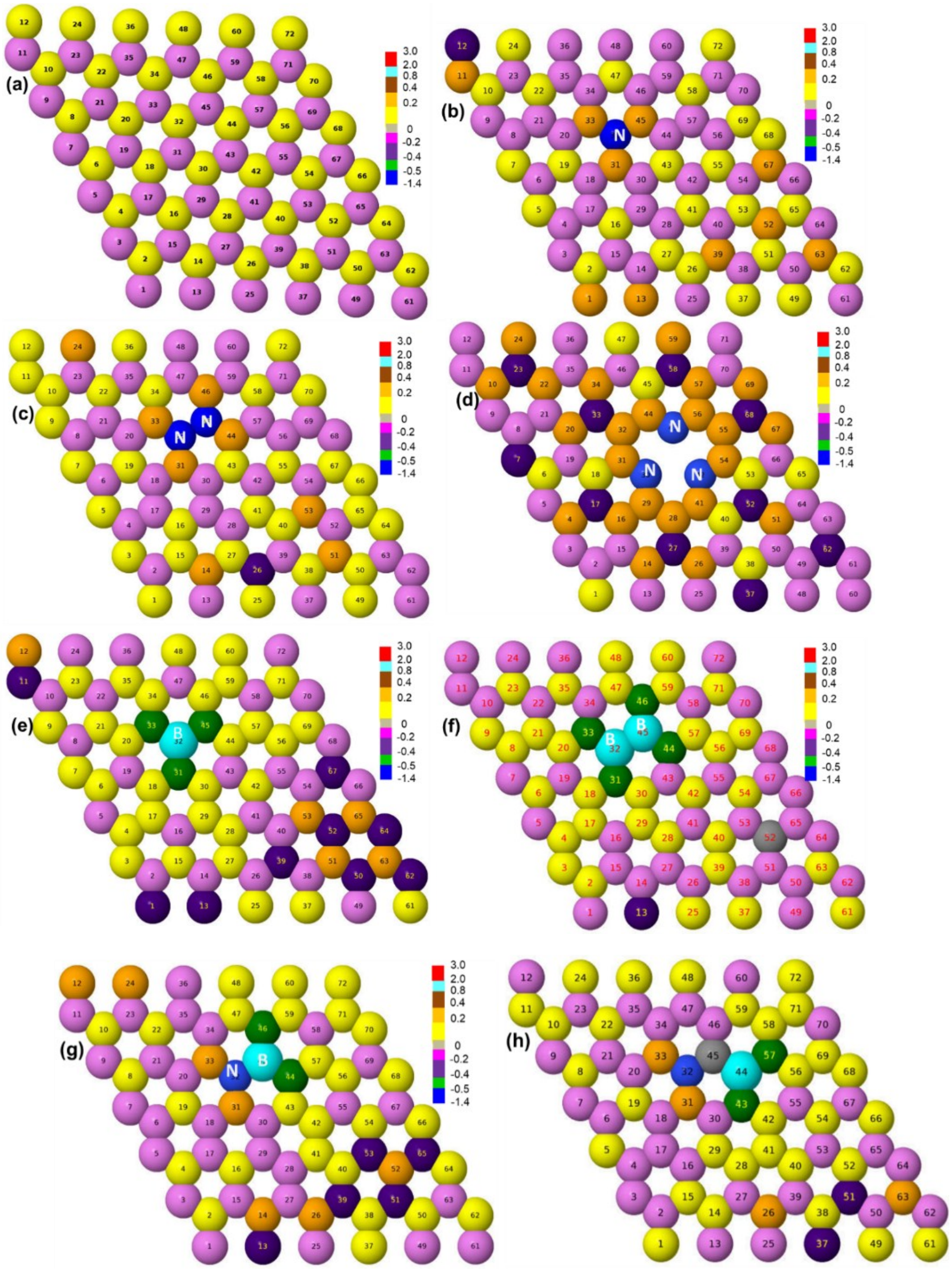

Figure S2: Bader effective charges of pure graphene (a), NG (b), 2NG (c), pyr–3NG (d), BG (e), 2BG (f), b-NBG (g), and s-NBG (h). The colors of the balls stand for relative values of charge density. The charge density decreases linearly from positive to negative values in the color order of red, cyan, pink, orange, yellow, gray, violet, indigo, green, and blue.

Doped atoms are labeled with their corresponding symbols and all the atoms are numbered showing on circles.

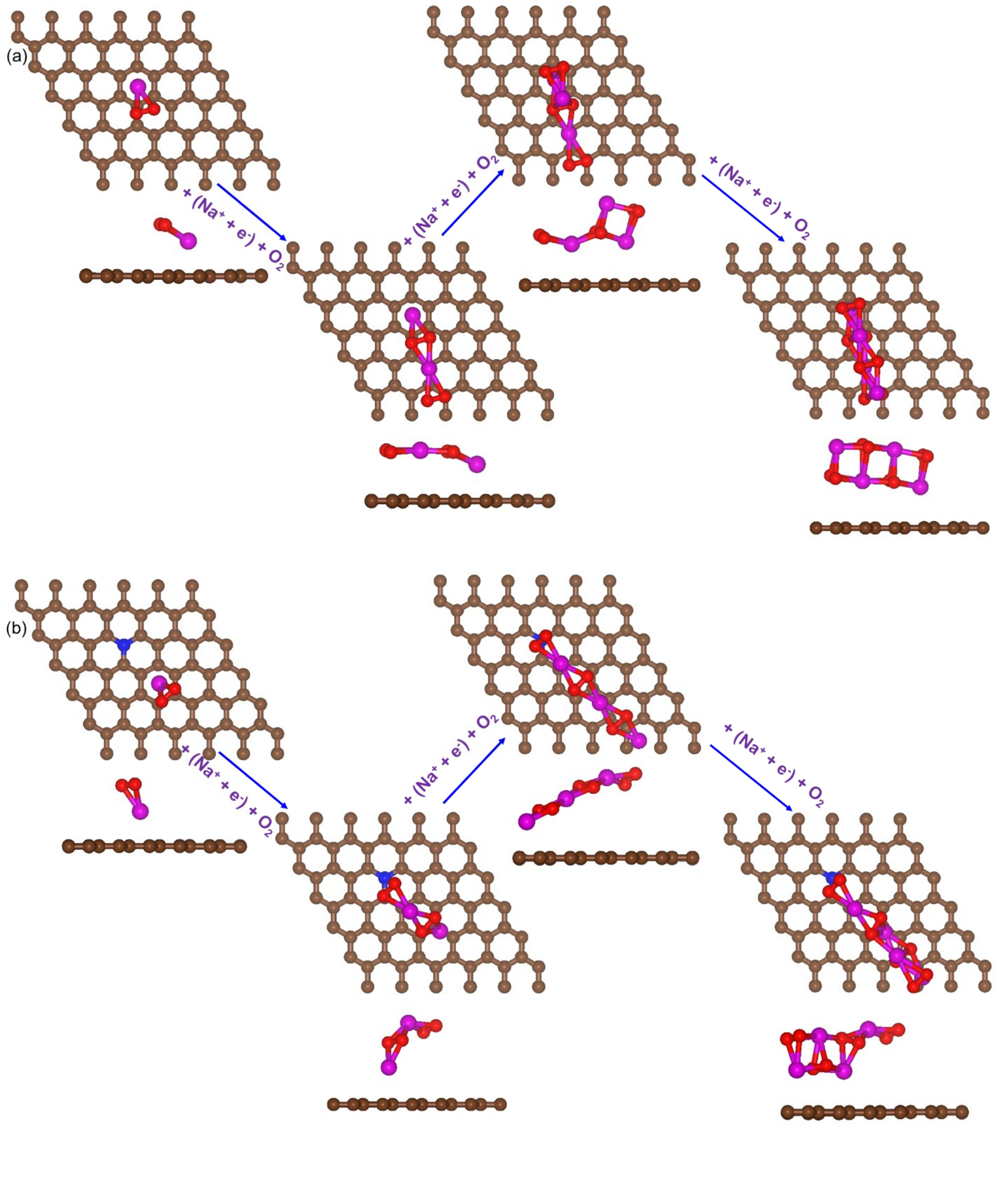

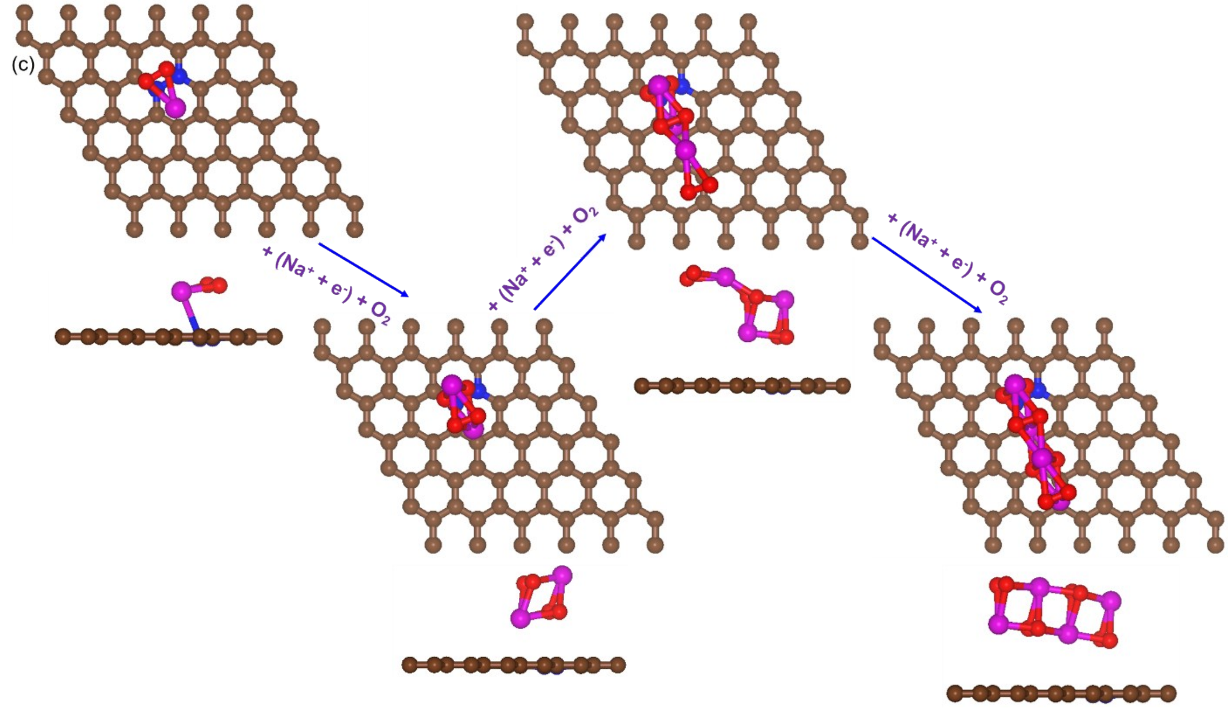

Figure S3: Schematics of the growing pathways of four formula unit of NaO_2 on pure graphene (a), NG (b) and 2NG (c). The gray and blue spheres indicate C and N atoms, respectively. Deposited atoms labeled as: Na (purple) and O (red).

Table S1: Bader charge analysis for the Na_4O_8 formed at the N doped graphene site.

	Transferred charge for the adsorbed last intermediate (Na_4O_8)												
	Na_1	Na_2	Na_3	Na_4	O_1	O_2	O_3	O_4	O_5	O_6	O_7	O_8	**Total**
Gr	0.90	0.89	0.89	0.90	-0.49	-0.50	-0.42	-0.43	-0.38	-0.36	-0.47	-0.44	**0.10**
NG	0.89	0.89	0.90	0.90	-0.53	-0.53	-0.41	-0.41	-0.36	-0.35	-0.50	-0.50	**0.00**
2NG	0.89	0.90	0.89	0.90	-0.44	-0.46	-0.51	-0.52	-0.38	-0.39	-0.55	-0.49	**-0.15**
pyr-3NG	0.88	0.90	0.90	0.90	-0.37	-0.36	-0.39	-0.41	-0.27	-0.26	-0.52	-0.50	**0.52**

Table S2: Bader charge analysis for the Na_4O_8 formed at the B doped graphene sites.

	Transferred charge for the adsorbed last intermediate (Na_4O_8)												
	Na_1	Na_2	Na_3	Na_4	O_1	O_2	O_3	O_4	O_5	O_6	O_7	O_8	**Total**
BG	0.89	0.89	0.90	0.91	-0.39	-0.38	-0.39	-0.39	-0.36	-0.37	-0.43	-0.43	**0.45**
2BG	0.88	0.89	0.91	0.90	-0.48	-0.52	-0.20	-0.26	-0.56	-0.56	-0.24	-0.25	**0.50**

Table S3: Bader charge analysis for the Na_4O_8 formed at the N, B-codoped graphene

	Transferred charge for the adsorbed last intermediate (Na_4O_8)												
	Na_1	Na_2	Na_3	Na_4	O_1	O_2	O_3	O_4	O_5	O_6	O_7	O_8	**Tot.**
b-NBG	0.89	0.89	0.9	0.91	-0.55	-0.44	-0.40	-0.42	-0.41	-0.42	-0.40	-0.27	**0.27**
s-NBG	0.89	0.89	0.9	0.91	-0.53	-0.43	-0.38	-0.40	-0.41	-0.40	-0.43	-0.31	**0.28**

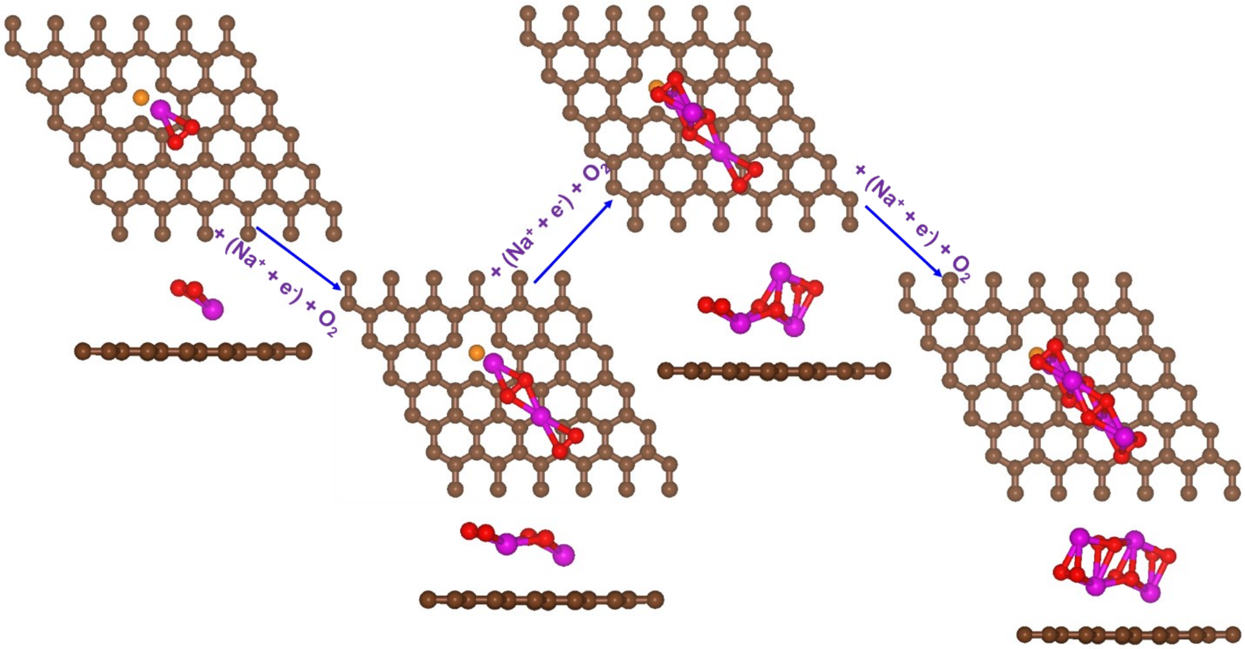

Figure S4: Schematics of the growing pathways of $4NaO_2$ on BG. The brown and orange spheres indicate C and B atoms, respectively. Deposited atoms labeled as: Na (purple) and O (red).

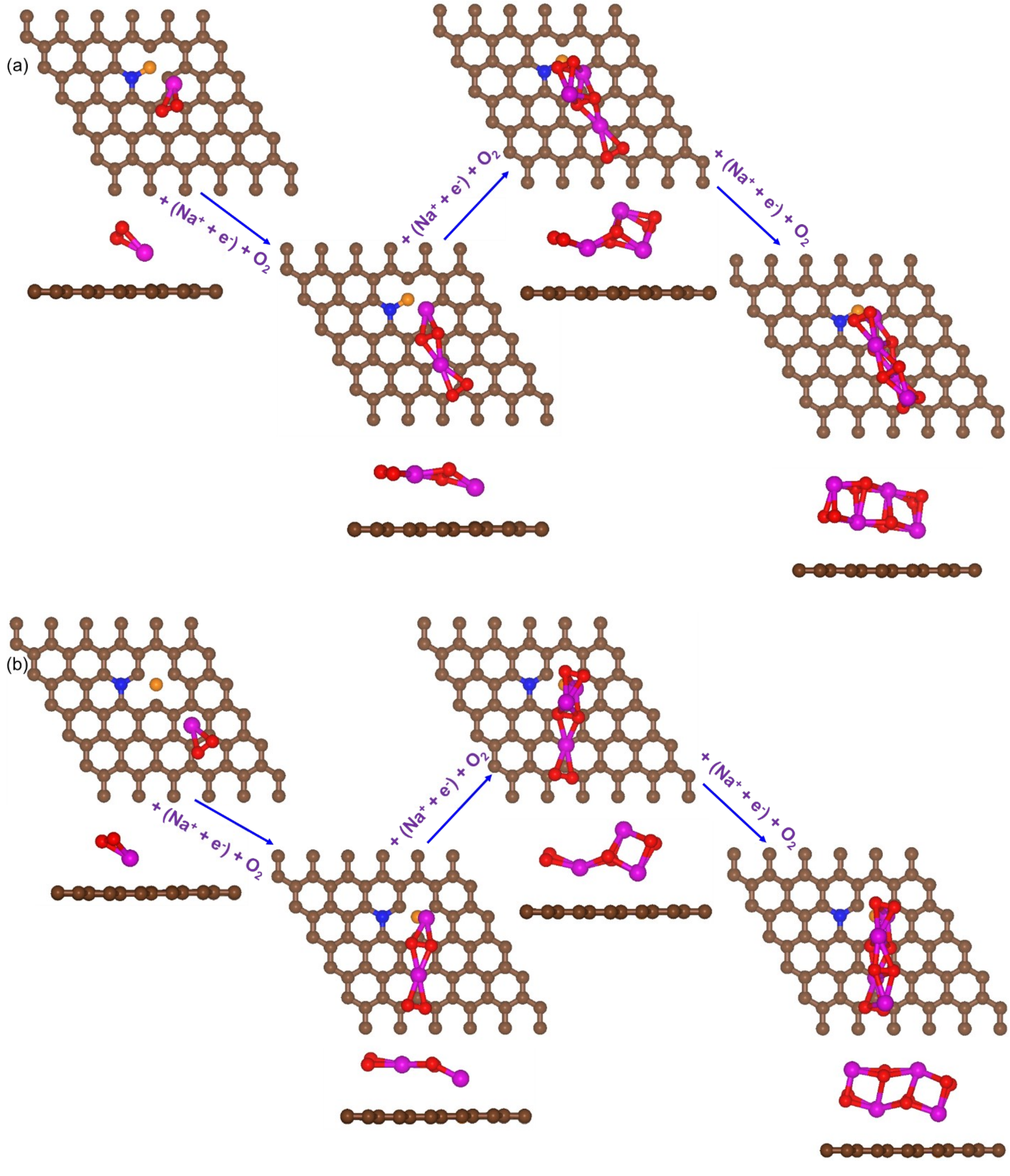

Figure S5: Schematics of the growing pathways of four formula unit of NaO_2 on b-NBG (a) and s-NBG (b). The brown, blue and orange spheres indicate C, N and B atoms, respectively. Deposited atoms labeled as: Na (purple) and O (red).

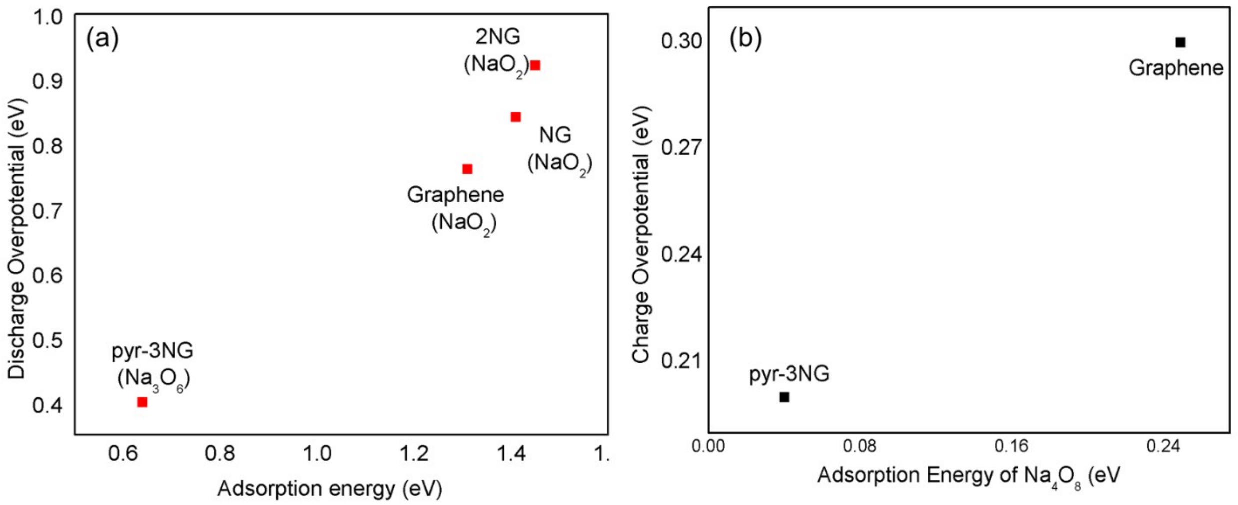

Figure S6: The η_{dis} as a function of the adsorption energy of NaO_2 (a) and the η_{ch} as a function of adsorption of Na_4O_8 (b) for N doped graphene structures

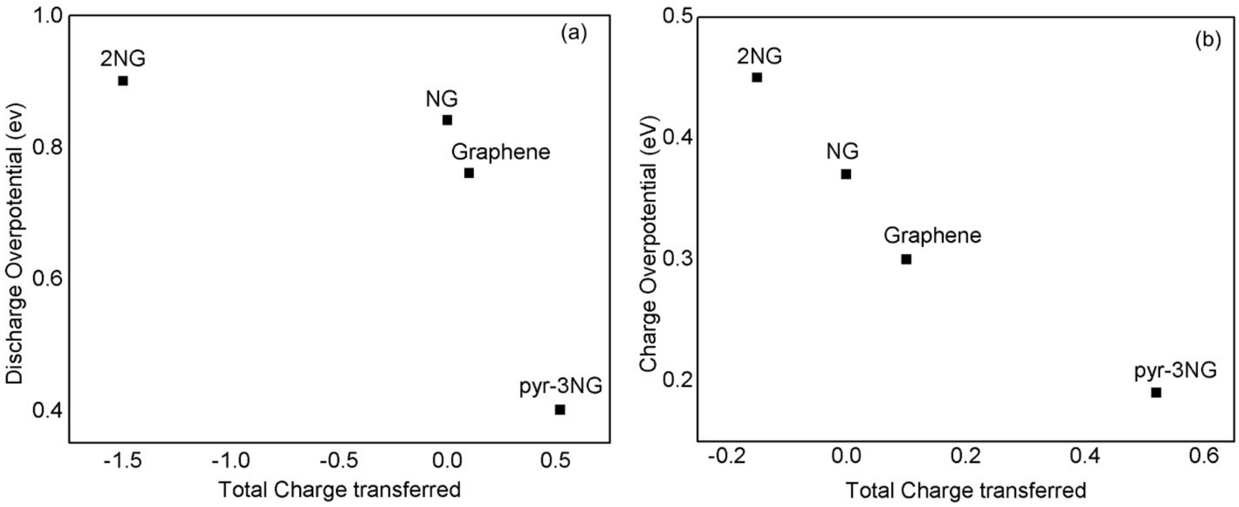

Figure S7: The η_{dis} as a function of the charge transferred of Na_4O_8 (a) and the η_{ch} as a function of charge transferred of Na_4O_8 (b) for N doped graphene structures

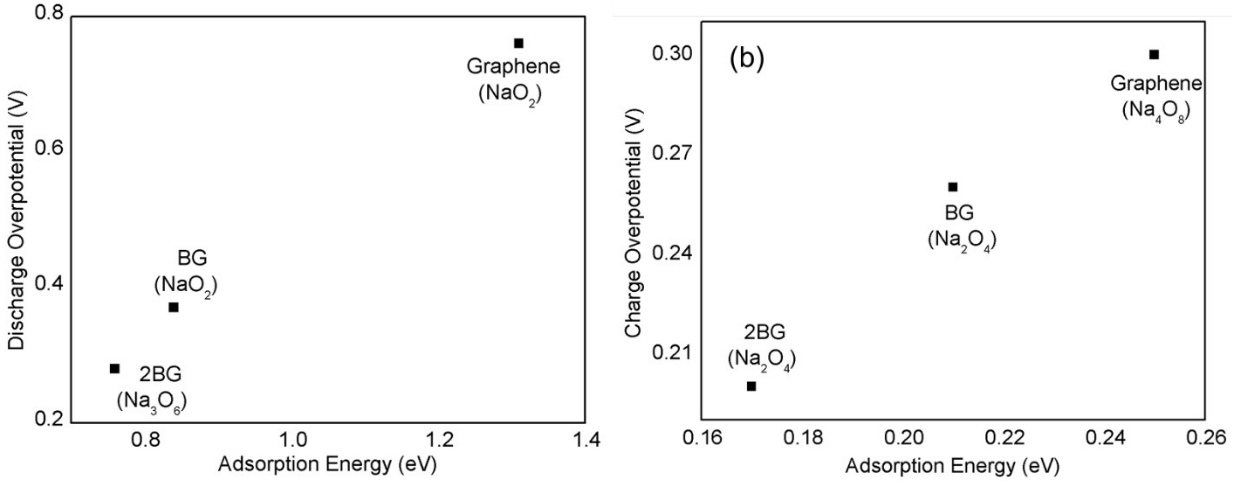

Figure S8: The η_{dis} as a function of the adsorption energy of NaO_2 (a) and the η_{ch} as a function of adsorption of Na_4O_8 (b) for B doped graphene structures

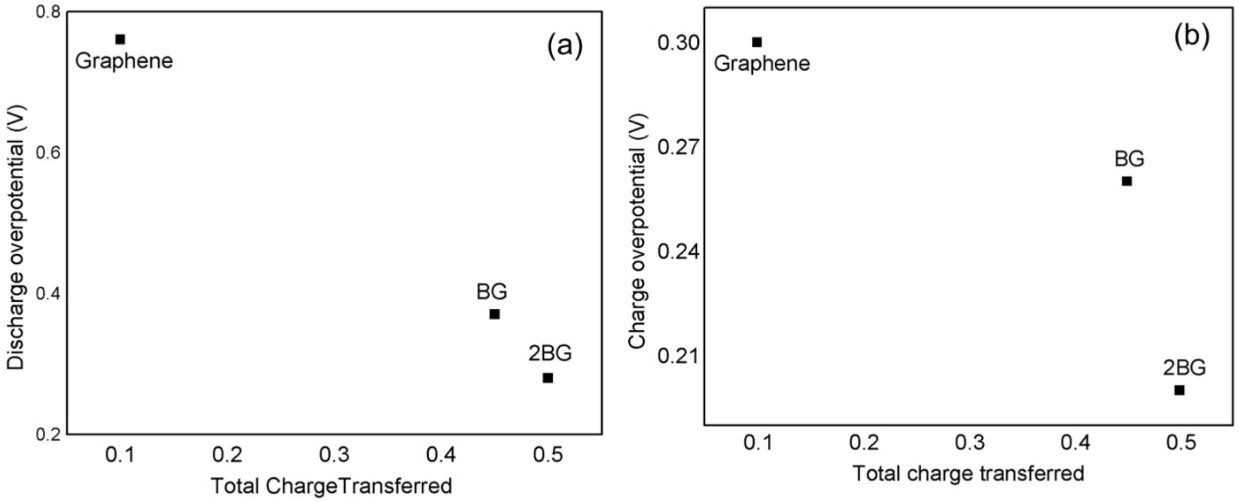

Figure S9: The η_{dis} as a function of the charge transferred of Na_4O_8 (a) and the η_{ch} as a function of charge transferred of Na_4O_8 (b) for B doped graphene structures

REFERENCE

1 W. Yin and Z. Fu, *ChemCatChem*, 2017, **9**, 1545–1553.
